# Efficacy of oseltamivir-peramivir combination therapy compared to oseltamivir monotherapy for *Influenza A* (H7N9) infection: a retrospective study

**DOI:** 10.1186/s12879-016-1383-8

**Published:** 2016-02-10

**Authors:** Yan Zhang, Hainv Gao, Weifeng Liang, Lingling Tang, Yida Yang, Xiaoxin Wu, Liang Yu, Ping Chen, Shufa Zheng, Huilin Ou, Lanjuan Li

**Affiliations:** The State Key Laboratory for Diagnosis and Treatment of Infectious Disease, Collaborative Innovation Center for Diagnosis and Treatment of Infectious Diseases, Department of Infectious Disease, The First Affiliated Hospital, College of Medicine, Zhejiang University, 310003 HangZhou, China

**Keywords:** *Influenza A*, H7N9 virus, Oseltamivir, Peramivir

## Abstract

**Background:**

Since the novel H7N9 avian influenza outbreak occurred in China in 2013, neuraminidase inhibitors (NAIs) such as oseltamivir and peramivir have been used as first-line drugs to treat the influenza virus infection. This study aimed to compare the efficacy of oseltamivir-peramivir combination therapy versus oseltamivir monotherapy.

**Methods:**

A retrospective study of 82 H7N9 confirmed patients was conducted by reviewing medical charts at the First Affiliated Hospital of ZheJiang University in China from April 1, 2013 to Feb 28, 2014. The patients’ clinical information was collected systematically, and we compared the virology and clinical data between oseltamivir monotherapy group (43 patients) and oseltamivir-peramivir combination group (39 patients).

**Results:**

The median duration from NAIs administration to H7N9 virus-negative in oseltamivir monotherapy group and oseltamivir-peramivir combination group was 6.50 and 7.00 days (*p* >0.05), respectively. The median decline of Day 2 to Day 0 (initiation of NAIs therapy) viral load was 0.00 and 0.69 log10 copies/μl (*p* >0.05) respectively in the monotherapy vs. combination therapy groups. The incidence of new Acute Respiratory Distress Syndrome during NAI administration was 63.89 and 77.78 % (*p* >0.05); while the mortality rates were 25.58 and 43.59 % (*p* >0.05) in the oseltamivir group vs. oseltamivir-peramivir group.

**Conclusions:**

Our results suggest that in adults with H7N9 virus infection, the use of oseltamivir-peramivir combination therapy was not superior to oseltamivir monotherapy.

## Background

Since the novel H7N9 avian influenza outbreak occurred in China in 2013, neuraminidase inhibitors (NAIs) such as oseltamivir and peramivir have been important and efficacious in curbing the viral infection [1]. From previous experience with influenza infections, the wide use of a single antiviral drug likely leads to drug resistance which can reduce the effectiveness of antiviral activity [2–6]. Furthermore, oseltamivir-resistant viral strains have been identified [7]. In order to decrease the emergence of other drug-resistant virus in the future, while at the same time ensure efficacious antiviral effects, new treatment strategies are required.

One such strategy was the combination of two NAIs to treat influenza infections. The basis of the combination therapy was that two or triple antiviral drugs may have additive synergistic effects and reduce drug resistance at the same time [2–6]. Combination of oseltamivir and peramivir showed additive to synergistic activity against *Influenza A* (H1N1) virus in vitro and in mice [8]. Another study showed possible additive to antagonistic effects in vitro [9]. In addition, a study carried out in mice showed the combination of oseltamivir and zanamivir therapy was not superior to zanamivir monotherapy [10]. Furthermore, a randomized double-blind and placebo-controlled clinical trial in adults with seasonal *Influenza A* H3N2 virus infection during 2008–2009 showed the oseltamivir-zanamivir combination therapy was not more effective than either oseltamivir or zanamivir monotherapy [11]. However, since the H7N9 virus has a different structure compared to H3N2, the effect of combination treatment versus monotherapy is unknown. Herein, we carried out a retrospective study to evaluate the efficacy of antiviral therapy of oseltamivir-peramivir combination compared to oseltamivir monotherapy in the treatment of adult patients with H7N9 virus infection.

## Methods

### Ethics statement

This study was approved by the First Affiliated Hospital of ZheJiang University ethics board.

### Patient enrollment

During the outbreak, patients with influenza symptoms onset (temperature ≥38.0 °C or at least one of respiratory symptoms including rhinorrhea, sore throat, cough, or nasal congestion), pneumonia of unknown origin, or patients who had recently been in close contact with birds or a H7N9-confirmed patient were screened in this study. Respiratory specimens (nasopharyngeal, oropharyngeal swabs or sputum) and blood samples were collected for H7N9 virus laboratory tests and conducted in these suspected cases. There were three methods for H7N9 laboratory diagnosis: real-time reverse-transcriptase-polymerase-chain-reaction assay (RT-PCR) assay, viral isolation, and H7N9 serological testing by modified hemagglutinin inhibition assay [12, 13]. The patients with laboratory diagnosis were defined as confirmed H7N9 patients [13]. Regardless of clinical severity, the confirmed H7N9 patients were admitted into the hospital and treated with NAIs.

This retrospective study was performed at the First Affiliated Hospital of ZheJiang University. Enrollment criteria included age ≥18 years with confirmed *Influenza A* (H7N9) virus infection, and acceptance of oral oseltamivir monotherapy or oral oseltamivir and intravenous peramivir combination therapy. All the 82 cases enrolled in the study were admitted during the study period from April 1, 2013 to Feb 28, 2014.

### Data collection

We reviewed medical charts and used standardized forms to gather information retrospectively. Clinical and laboratory information was collected systematically from admission to discharge for every patient, including demographic information, baseline and follow-up clinical information.

After admission, respiratory specimens (nasopharyngeal swabs, sputum, or endotracheal aspirates) were collected daily to determine H7N9 viral RNA by PCR analysis. The second negative result of two respiratory samples collected in two-consecutive days was considered the time to stop NAIs therapy and represented an undetectable viral RNA level. We defined the duration between NAI administration and undetectable viral RNA level as the time from NAI administration to virus-negative. As we could not determine the exact virus infection time, following another report [14], we defined the interval between symptom onset and the date of the first negative result of two consecutive respiratory samples as the RNA shedding. Severity of illness was evaluated according to the Acute Physiology and Chronic Health Evaluation (APACHE) II score on the day of admission. Moderate-to-severe Acute Respiratory Distress Syndrome (ARDS) as diagnosed by the ARDS Berlin definition, i.e. severe hypoxemia (PaO_2_/ FiO_2_ ≤200 mmHg with PEEP ≥5 cm H_2_O), associated with bilateral opacities on chest X-ray, which could not be fully explained by cardiac failure or fluid overload.

### Outcome measures

The primary outcome was the time from NAI administration to virus-negative. The second outcome was the decline of virus load (measured by log10 virus load) between Day 0 (the day NAI therapy was initiated) and Day 2 in patients with confirmed H7N9 virus infection. Based on the viral shedding kinetics in seasonal influenza patients treated by NAIs, the Day 2 viral outcome seemed the most suitable to evaluate virology effects [15, 16]. The clinical end points included the incidence of ARDS after NAIs administration and in-hospital mortality.

### Statistical analysis

Categorical variables were calculated by frequency analysis. The numerical variables of normal distributions was represented by means (±standard deviations), abnormal distributions was represented by medians (interquartile, IQR). Two sample Student’s t test was performed to assess the significance of the time from NAI administration to H7N9 virus-negative, the decline of log10 virus load between Day 0 and Day 2. Chi-square test was performed to compare the rate of new ARDS after NAI administration and the total in-hospital mortality. All analyses were two-tailed and the *P* value <0.05 was considered significant. All analyses were conducted using SPSS for Windows (version 16.0).

## Results

### Study population characteristics

From April 1, 2013 to Feb 28, 2014, about 1950 patients were tested for H7N9 viral infection, and 87 patients were positive. Five of them received other antiviral drugs other than oseltamivir or peramivir. Therefore, a total of 82 adult patients were enrolled in our study. The mean age was 58.21 years (±14.31) and 68.29 % (56 patients) were male. Forty four (53.66 %) patients had one or more coexisting conditions. Hypertension, diabetes, coronary heart disease were the most common coexisting conditions (Table 1). The most common symptom was fever (82 patients, 100 %), followed by cough (73 patients, 89.02 %), Sputum production (52 patients, 63.41 %), and shortness of breath (52 patients, 63.41 %) (Table 1).

**Table 1:** Demographic and clinical characteristics of 82 confirmed H7N9 patients

Characteristic		No. of cases (%)		*P* value*
	Total (*n* = 82)	The first wave (*n* = 39)	The second wave (*n* = 43)	
Male patients	56 (68.29)			
Age (years)				
18–49	19(23.17)	8(20.51)	11(25.58)	0.09
50–64	35(42.68)	13(33.33)	22(51.16)	
≥65	28(34.15)	18(46.15)	10(23.26)	
Coexisting condition				
Any	44(53.66)	23(84.62)	21(48.84)	0.38
Hypertension	38(46.34)			
Diabetes	17(20.73)			
Coronary heart disease	6(7.31)			
Cerebrovascular disease	4(4.86)			
Chronic obstructive pulmonary disease	3(3.65)			
Cancers^a^	2(2.43)			
Immunosuppression^b^	2(2.43)			
Hepatitis B infection	1(1.22)			
Pregnancy	1(1.22)			
Symptoms				
Fever	82(100)			
Cough	73(89.02)			
Sputum production	52(63.41)			
Shortness of breath	52(63.41)			
Fatigue/weekness	32(39.02)			
Hemoptysis	17(20.73)			
Gastrointestinal symptom^c^	11(3.41)			

^a^Cancers included leukemia and lymphoma

^b^Immunosuppression caused by the immunosuppressive drug after renal transplantation

^c^Gastrointestinal symptom included nausea, vomiting, diarrhea, and abdominal pain

*The p value was calculated between the first and second H7N9 wave

All the patients received NAIs therapy after admission. The median time from symptom onset to NAIs therapy was 6.00 days (interquartile range, 4 to 8). Eight patients (9.76 %) received NAIs therapy within 48 h after the symptom onset, and 35 patients (42.68 %) within 5 days, 47 patients (57.31 %) more than 5 days. Forty two patients (51.22 %) received glucocorticoid therapy, including intravenous methylprednisolone and intravenous dexamethasone. There were 41 (50 %) patients developed bacterial or fungal infection or colonisation by positive sputum culture during hospitalization, including *Acinetobacter baumannii*, *Klebsiella pneumoniae*, *Staphylococcus aureus*, *Burkholderia cepacia* and C*andida albicans*.

Among the 82 patients, 39 patients (47.56 %) were enrolled in the first H7N9 wave from April 1, 2013 to May 31, 2013, and 43 patients (52.43 %) were enrolled in the second wave from Nov 30, 2013 to Feb 28, 2014. In the first H7N9 wave, the median age was 63 years(IQR, 55 to 70) and 46.15 % (18 patients) were ≥65 years, while in the second wave, the median age was 57 years(IQR, 47 to 64) and 51.16 %(22 patients,) were 50–64 years. Compared with the first wave, more patients in the second wave had no coexisting condition (Table 1). The rates of ARDS were respectively 27/39 (69.23 %) and 33/43 (76.74 %), in-hospital mortality were respectively 9/39 (23.08 %) and 19/43 (44.19 %) in the first and second wave of H7N9.

Based on the different NAIs drugs they accepted, the 82 patients were divided into oseltamivir monotherapy therapy group (O, *n* = 43, with 39 cases enrolled in the H7N9 first wave, four cases in the second wave) and oseltamivir-peramivir combination therapy group (OP, *n* = 39, all enrolled in the second H7N9 wave).

In the oseltamivir monotherapy group, 23 patients (53.49 %) were treated with oseltamivir dosage of 75 mg orally twice daily, 19 patients (44.19 %) were treated with 150 mg twice daily, and one patient (2.33 %) received 75 mg once. In the oseltamivir-peramivir combination therapy group, 24 patients (61.54 %) were treated with 75 mg oseltamivir twice and 600 mg peramivir once daily; five patients (12.82 %) were treated with 75 mg oseltamivir twice and 300 m g peramivir once daily; six patients (15.38 %) were treated with 150 mg oseltamivir twice and 600 mg peramivir once daily; two patients (5.13 %) were treated with 150 mg oseltamivir twice and 300 mg peramivir once daily; one patient (2.56 %) changed oseltamivir dosage to 150 mg twice daily after 10 days of 75 mg twice daily with 600 mg peramivir once daily; one patient (2.56 %) changed oseltamivir dosage to 75 mg twice daily after 6 days of 75 mg once daily in the local hospital with 600 mg peramivir once daily.

### Endpoints

#### Primary outcome: duration from NAIs therapy to H7N9 virus negative

Among the 82 patients, there were seven patients whose H7N9 virus status was positive till death in the oseltamivir-peramivir group, and five in oseltamivir monotherapy group. Excluding these 12 patients, the oseltamivir monotherapy group had 38 (88.37 %) patients and the oseltamivir-peramivir group had 32 (82.05 %) patients. The median time from NAIs therapy to negative viral RNA shedding of the remaining 70 patients was 7.00 days (IQR, 5 to 9). The median duration of viral shedding in the whole 82 patients was 12.00 days (IQR, 9.75 to 17), in the oseltamivir monotherapy group was 11 days (IQR, 9 to 16.25), and in the oseltamivir-peramivir combination group was 13 days (IQR, 11 to 17). Baseline information of the two groups is listed in Table 2. There was no difference between the two groups in regard to age, gender, the time from symptom onset to antivirus treatment, the viral load before NAIs treatment and the APACHE II score before NAIs therapy (*p* >0.05). The median time from antiviral treatment to viral-negative in oseltamivir monotherapy group was 6.50 days (IQR, 4 to 8), and oseltamivir-peramivir combination therapy group was 7.00 days (IQR, 6 to 9.75) (*p* >0.05; Table 2). A total of seven patients in the oseltamivir monotherapy group and 11 patients in the oseltamivir-peramivir combination therapy group died after their H7N9 status was viral-negative.

**Table 2:** Baseline information and the duration from NAIs therapy to H7N9 virus-negative

Patients	Characteristics	O + P	O	*P* Value
All patients		*n* = 39	*n* = 43	
Patients whose virus still positive till death		*n* = 7	*n* = 5	
Study patients		*n* = 32	*n* = 38	
	Age (years): mean (SD)	55.22(14.25)	60.21(14.89)	0.64
	No. of male (%)	22(68.75 %)	27(71.05 %)	1.00
	Time from symptom onset to NAIs administration (days):median (IQR)	7.00(4.25, 8.75)	5.00(4.00, 7.00)	0.52
	APACHE II score: mean(SD)	17.47(8.08)	20.82(8.17)	0.87
	Viral load(log10 /ul) at day 0: median(IQR)	3.30(2.91, 4.1)	3.29(2.70, 4.47)	0.12
	Duration from NAIs taken to H7N9 virus negative(days): median(IQR)	7.00(6.00, 9.75)	6.50(4.00, 8.00)	0.67

*O* oseltamivir monotherapy, *O+P* oseltamivir-peramivir combination therapy, *IQR* interquartile range, percentile 25 – percentile75

#### Secondary outcome: decline of log10 virus load between Day 0 and Day 2

Among the 82 confirmed patients, there were 60 patients with both Day 0 and Day 2 viral data, comprising 31 patients in the oseltamivir-peramivir combination therapy group, and 29 patients in oseltamivir monotherapy group. The median viral load decrease of Day 2 to Day 0 was 0.69 log10 copies/μl (IQR,0.31 to 1.56) in the oseltamivir-peramivir combination therapy group, and 0.00 log10 copies/μl (IQR, −0.59 to 1.18) in the oseltamivir monotherapy group (*p* >0.05; Table 3).

**Table 3:** Baseline information and the decrease of log10 virus load between Day 0 and Day 2

Patients	Characteristics	O+P group	O group	*P* value
All patients		*n* = 39	*n* = 43	
Patients with both day 0 and day 2 available specimens		*n* = 31	*n* = 29	
	Age (years): mean (SD)	55.45(14.80)	60.79(14.62)	0.94
	No. of male (%)	22(56.41)	22(51.16)	0.45
	Time from symptom onset to NAIs administration (days):median(IQR)	7.00(5.00, 8.00)	5.00(4.00, 7.00)	0.46
	APACHE II score: mean(SD)	18.53(8.83)	22.69(8.53)	0.83
	Viral load decrease between day 2 and day 0 (log10 /ul): median(IQR)	0.69(0.31, 1.56)	0.00(−0.59, 1.18)	0.06

*O* oseltamivir monotherapy, *O+P* oseltamivir-peramivir combination therapy, *IQR* interquartile range, percentile 25 – percentile75

#### Clinical outcome: the incidence of new Acute Respiratory Distress Syndrome after NAIs administration and in-hospital mortality

Of the total 82 patients, 7 (18.42 %) patients had ARDS before receiving NAIs treatment in the oseltamivir monotherapy group, and 3 (9.10 %) patients in the oseltamivir-peramivir combination therapy group. These 10 patients were therefore excluded from this portion of the study, and 36 patients remained in each treatment group. The incidence of newly-developed ARDS during NAI therapy was 23/36 (63.89 %) in oseltamivir monotherapy group and 28/36 (77.78 %) in the oseltamivir-peramivir combination therapy group (*p* >0.05; Table 4).

**Table 4:** Baseline and the incidence of ARDS

Patients	Characteristics	O+P group	O group	*P* value
All patients		*n* = 39	*n* = 43	
patients had already got ARDS before received NAIs		*n* = 3	*n* = 7	
Study patients		*n* = 36	*n* = 36	
	Age (years): mean (SD)	52.58(12.70)	59.31(13.85)	0.44
	No. of male (%)	24	24	1.00
	Time from symptom onset to NAIs administration (days):median(IQR)	7.00(4.25, 8.00)	6.00(4.00,8.00)	0.57
	Viral load(log10 /ul) at day 0: median(IQR)	4.07(3.00, 4.47)	3.58(2.89,4.47)	0.73
	APACHE II score: mean(SD)	19.17(8.28)	20.22(7.76)	0.58
	New ARDS developed patients (%)	28(77.78)	23(63.89)	0.30

*O* oseltamivir monotherapy, *O+P* oseltamivir-peramivir combination therapy, *IQR* interquartile range, percentile 25 – percentile75

In the 82 patients, the overall in-hospital mortality was 43.59 % in oseltamivir-peramivir combination therapy group, and 25.58 % in oseltamivir monotherapy group (Table 5).

**Table 5:** Baseline information and in-hospital mortality

Patients	Characteristics	O+P group	O group	*P* value
all patients included in the study *n* = 82		*n* = 39	*n* = 43	
	Age (years): mean (SD)	56.51(13.86)	59.74(14.71)	0.53
	No. of male (%)	27(69.23 %)	29(67.44 %)	1.0
	Time from symptom onset to NAIs administration (days):median(IQR)	7.00(5.00,8.00)	5.00(4.00,7.00)	0.16
	APACHE II score: mean(SD)	19.05(8.41)	21.09(8.33)	0.86
	Viral load(log10 /ul) at day 0: median(IQR)	3.34(3.06, 4.45)	3.53(2.84, 5.07)	0.08
	Mortality(%)	17(43.59)	11(25.58)	0.11

*O* oseltamivir monotherapy, *O+P* oseltamivir-peramivir combination therapy, *IQR* interquartile range, percentile 25 – percentile75

## Discussion

Our retrospective study examined the effect of oseltamivir-peramivir combination antiviral therapy in H7N9 influenza, as compared to oseltamivir monotherapy. The results showed that the oseltamivir-peramivir combination therapy not apparently superior to oseltamivir monotherapy in the adults with H7N9 virus infection during April 1, 2013 to Feb 28, 2014 in China. To the best of our knowledge, ours is the first study to compare the efficacy of oseltamivir-peramivir combination therapy and oseltamivir monotherapy on the H7N9 virus.

Most in vitro studies that assess the efficacy of combination NAIs therapy have shown synergism and additive effects. The combination of antivirals not only decreases the emergence of resistant strains, but also ensures antiviral effect [2–6]. Therefore, the combination of oseltamivir and peramivir was hypothesized to be more effective. However, our investigation showed that oseltamivir-peramivir combination was not superior to oseltamivir monotherapy. In other words, the combination did not lead to additive effects at least. Our findings are in agreement with a clinical trial that showed a lack of additive or synergistic effect between oseltamivir and zanamivir for seasonal *Influenza A* H3N2 virus during the 2008–2009 season [11], and a randomized trial that found no difference between oseltamivir-zanamivir combination and oseltamivir alone for *Influenza A* (H1N1)pdm09 virus [17]. Oseltamivir and peramivir are both neuraminidase inhibitors with similar mechanisms of action. Synergy or additive effects generally occur in antiviral drugs with different mechanisms of action [8]. Malaisre et al. reported that compared oseltamivir, peramivir acts on neuraminidase N1, and found that peramivir had a tighter binding to neuraminidase N1 than oseltamivir [18], and antagonistic interactions may exist. Therefore, further research is needed to investigate whether peramivir has a tighter binding to neuraminidase N9 which could prevent the action of oseltamivir.

The patients initiated NAI therapy a relatively long time after the onset of influenza symptoms (median 6.00 days, IQR, 4 to 8). Wiku Adisasmito et al. found that H5N1 patients could still benefit when initiated oseltamivir up to 6–8 days after onset of symptoms [19]. Here, the patients who initiated NAI treatment more than 5 days after onset of symptoms remained enrolled in the current study. However, the timing of NAI initiation was important. Early treatment was related to better outcome. Jain et al. showed the administration of antiviral drugs within two days of the onset of symptoms was the only independent risk factor affecting the prognosis of H1N1 [20]. The Centers for Disease Control and Prevention of the United States (CDC) recommends the initiation of antiviral drugs within 5 days of onset of symptoms for H1N1 infections. Regarding H7N9 virus, delayed antiviral therapy may be associated with more severe illness [21]. Gao HN et al. pointed out that the risk of death increased when antiviral therapy was initiated in more than 5 days after onset of symptoms [22]. In the current study, as many as 47.56 % patients (39) initiated NAIs in more than 5 days after onset of symptoms. The delayed administration of NAIs might affect the therapeutic effect of NAIs. There might be a difference between oseltamivir-peramivir combination therapy and oseltamivir monotherapy when administered within 48 h or 5 days after symptom onset. Smee et al. reported that oseltamivir-peramivir combination performed better than either monotherapy in vitro and in mice infected with *Influenza A*/NWS/33 (H1N1) virus [8]. In that study, the drugs were administered immediately after viral infection.

In the current study, most patients in the oseltamivir monotherapy group (39 patients, 90.70 %) were enrolled in the first H7N9 wave, while all the patients in the oseltamivir-peramivir combination group were enrolled in the second H7N9 wave. It was observed that H7N9 virus in the second wave to infect younger patients with less concomitant disease. The second wave had higher mortality. The influenza virus is an RNA virus, with a high error rate during transcription [23]. The oseltamivir-resistance is more likely to emerge in severe cases [24], such as H7N9 cases. During the first H7N9 wave in China, oseltamivir-resistant H7N9 virus had already emerged. NA-E119V, NA-I222K, and NA-I222R reduced inhibition by oseltamivir, and NA-R292K caused highly reduced inhibition by oseltamivir and peramivir [7]. The NA-R292K mutation has been reported to emerge within 2 days of administration of oseltamivir, and it is associated with the poor clinical outcome [1]. Corticosteroid therapy seems to be a risk factor [1]. The widespread use of oseltamivir and corticosteroid by the current patients may have caused a mutation to emerge. However, the H7N9 viral RNA was not sequenced for these 82 cases, so it was not possible to know whether there were any mutations associated with resistance to NAIs and the difference in H7N9 viral sequence between two groups. Further research is needed.

In the current study, the dosage of NAI varied widely. The majority of the patients (38 patients, 94.44 % in the oseltamivir monotherapy group; 42 patients, 97.67 % in the oseltamivir-peramivir combination group) received the standard or double dosing of NAIs. Two oseltamivir dose-comparison studies in influenza showed no difference from standard dosage (75 mg twice daily) or double dosage (150 mg twice daily) in virological clearance or clinical outcome [25, 26]. One hypothesis is that the action of oseltamivir may be saturable [26]. Previous studies indicate that doubling oseltamivir dosage had no effect on viral clearance or clinical outcome. However, for oseltamivir-peramivir combination therapy, the proportion of the two drugs may influence the result. Nguyen et al. compared the combinations of zanamivir and peramivir or oseltamivir in vitro and found a concentration-related additive to antagonistic effects for H1N1 viruses [9]. The combination of different concentrations of oseltamivir and peramivir for *Influenza A* (H1N1) virus in vitro and in mice showed additive to synergistic effects [8]. In this way, the various combination ratios may affect the result. Suitable concentration ratios may be necessary to produce additive or synergistic effects when treating H7N9 patients with combinations of oseltamivir and peramivir.

The current study suggested that the median duration of viral shedding of H7N9 virus was 12 days (IQR, 9.75 to 17). The viral load of the seasonal influenza usually shed for 5 days in adults [27, 28]. The 2009 H1N1 shed for a median of 5 days (3 to 6) [14]. The current results showed that H7N9 could be shed for a longer period of time than 2009 H1N1 and seasonal influenza. A study showed that a longer viral shedding interval to be related to severe illness in H7N9 patients [21]. Similar findings were observed in influenza A(H1N1) patients [1]. In influenza H1N1, the early treatment within 2 days of onset of symptoms could reduce the duration viral RNA shedding [14]. Studies have reported that delayed treatment with oseltamivir may be related to delayed viral clearance [26]. Prolonged positive viral RNA had been shown to correlate with morbidity in hospital [26]. In the current study, the delayed administration of NAIs might contribute to the high morbidity. Therefore, the early initiation of NAIs is recommended.

There are several limitations to the current study. First, it was a retrospective study and the sample size was too small for robust investigation in subgroups of patients. Despite the use of a standardized case-report form, not all information was available for all 82 patients. Secondly, the H7N9 viral RNA of these 82 cases was not sequenced for analysis of antiviral resistance. It was not possible to determine whether there were any mutations associated with resistance to NAIs in either group, especially the NA-R292K. Third, the dosage of oseltamivir and peramivir varied widely. The heterogeneity may influence the antiviral effect of two groups. Fourthly, most people in our study received NAIs more than 5 days, not the optimal time. It may influence the effect of the NAIs. Further prospective clinical studies should be performed to compare the effect between NAI monotherapy and combination therapy with two NAIs with standardization NAI administration. And the NAI-resistance should be analyzed.

## Conclusions

In conclusion, our study has shown that oseltamivir-peramivir combination was not superior to oseltamivir monotherapy for treating H7N9 influenza; therefore the use of the combination therapy may not be useful when treating critically ill patients.

## Abbreviations

APACHE: acute physiology and chronic health evaluation
ARDS: acute respiratory distress syndrome
CDC: Centers for Disease Control and Prevention of American
IQR: interquartile range
NAIs: neuraminidase inhibitors
O: oseltamivir
P: peramivir
RT-PCR: real-time reverse-transcriptase-polymerase-chain-reaction assay

## References

[1] Hu Y, Lu S, Song Z, Wang W, Hao P, Li J (2013). Association between adverse clinical outcome in human disease caused by novel influenza A H7N9 virus and sustained viral shedding and emergence of antiviral resistance. Lancet (London, England).

[2] Galabov AS, Simeonova L, Gegova G (2006). Rimantadine and oseltamivir demonstrate synergistic combination effect in an experimental infection with type A (H3N2) influenza virus in mice. Antivir Chem Chemother.

[3] Ilyushina NA, Hay A, Yilmaz N, Boon AC, Webster RG, Govorkova EA (2008). Oseltamivir-ribavirin combination therapy for highly pathogenic H5N1 influenza virus infection in mice. Antimicrob Agents Chemother.

[4] Ilyushina NA, Hoffmann E, Salomon R, Webster RG, Govorkova EA (2007). Amantadine-oseltamivir combination therapy for H5N1 influenza virus infection in mice. Antivir Ther.

[5] Smee DF, Bailey KW, Morrison AC, Sidwell RW (2002). Combination treatment of influenza A virus infections in cell culture and in mice with the cyclopentane neuraminidase inhibitor RWJ-270201 and ribavirin. Chemotherapy.

[6] Smee DF, Hurst BL, Wong MH, Bailey KW, Morrey JD (2009). Effects of double combinations of amantadine, oseltamivir, and ribavirin on influenza A (H5N1) virus infections in cell culture and in mice. Antimicrob Agents Chemother.

[7] Marjuki H, Mishin VP, Chesnokov AP, Jones J, De La Cruz JA, Sleeman K (2015). Characterization of drug-resistant influenza A(H7N9) variants isolated from an oseltamivir-treated patient in Taiwan. J Infect Dis.

[8] Smee DF, Hurst BL, Wong MH, Tarbet EB, Babu YS, Klumpp K (2010). Combinations of oseltamivir and peramivir for the treatment of influenza A (H1N1) virus infections in cell culture and in mice. Antivir Res.

[9] Nguyen JT, Hoopes JD, Le MH, Smee DF, Patick AK, Faix DJ (2010). Triple combination of amantadine, ribavirin, and oseltamivir is highly active and synergistic against drug resistant influenza virus strains in vitro. PLoS One.

[10] Pizzorno A, Abed Y, Rheaume C, Boivin G (2014). Oseltamivir-zanamivir combination therapy is not superior to zanamivir monotherapy in mice infected with influenza A(H3N2) and A(H1N1)pdm09 viruses. Antivir Res.

[11] Duval X, van der Werf S, Blanchon T, Mosnier A, Bouscambert-Duchamp M, Tibi A (2010). Efficacy of oseltamivir-zanamivir combination compared to each monotherapy for seasonal influenza: a randomized placebo-controlled trial. PLoS Med.

[12] Gao R, Cao B, Hu Y, Feng Z, Wang D, Hu W (2013). Human infection with a novel avian-origin influenza A (H7N9) virus. N Engl J Med.

[13] Li Q, Zhou L, Zhou M, Chen Z, Li F, Wu H (2014). Epidemiology of human infections with avian influenza A(H7N9) virus in China. N Engl J Med.

[14] Yu H, Liao Q, Yuan Y, Zhou L, Xiang N, Huai Y (2010). Effectiveness of oseltamivir on disease progression and viral RNA shedding in patients with mild pandemic 2009 influenza A H1N1: opportunistic retrospective study of medical charts in China. BMJ (Clinical Research Ed).

[15] Hayden FG, Osterhaus AD, Treanor JJ, Fleming DM, Aoki FY, Nicholson KG (1997). Efficacy and safety of the neuraminidase inhibitor zanamivir in the treatment of influenzavirus infections. GG167 Influenza Study Group. N Engl J Med.

[16] Treanor JJ, Hayden FG, Vrooman PS, Barbarash R, Bettis R, Riff D (2000). Efficacy and safety of the oral neuraminidase inhibitor oseltamivir in treating acute influenza: a randomized controlled trial. US Oral Neuraminidase Study Group. JAMA.

[17] Escuret V, Cornu C, Boutitie F, Enouf V, Mosnier A, Bouscambert-Duchamp M (2012). Oseltamivir-zanamivir bitherapy compared to oseltamivir monotherapy in the treatment of pandemic 2009 influenza A(H1N1) virus infections. Antivir Res.

[18] Bantia S, Arnold CS, Parker CD, Upshaw R, Chand P (2006). Anti-influenza virus activity of peramivir in mice with single intramuscular injection. Antivir Res.

[19] Adisasmito W, Chan PK, Lee N, Oner AF, Gasimov V, Aghayev F (2010). Effectiveness of antiviral treatment in human influenza A(H5N1) infections: analysis of a Global Patient Registry. J Infect Dis.

[20] Jain S, Benoit SR, Skarbinski J, Bramley AM, Finelli L (2012). Influenza-associated pneumonia among hospitalized patients with 2009 pandemic influenza A (H1N1) virus--United States, 2009. Clin Infect Dis.

[21] Leung YH, To MK, Lam TS, Yau SW, Leung OS, Chuang SK. Epidemiology of human influenza A(H7N9) infection in Hong Kong. J Microbiol Immunol Infect. 2015;S1684-1182(15)00772-0. 10.1016/j.jmii.2015.06.004 26220305

[22] Gao HN, Lu HZ, Cao B, Du B, Shang H, Gan JH (2013). Clinical findings in 111 cases of influenza A (H7N9) virus infection. N Engl J Med.

[23] Stech J, Xiong X, Scholtissek C, Webster RG (1999). Independence of evolutionary and mutational rates after transmission of avian influenza viruses to swine. J Virol.

[24] Petersen E, Keld DB, Ellermann-Eriksen S, Gubbels S, Ilkjaer S, Jensen-Fangel S (2011). Failure of combination oral oseltamivir and inhaled zanamivir antiviral treatment in ventilator- and ECMO-treated critically ill patients with pandemic influenza A (H1N1)v. Scand J Infect Dis.

[25] Lee N, Hui DS, Zuo Z, Ngai KL, Lui GC, Wo SK (2013). A prospective intervention study on higher-dose oseltamivir treatment in adults hospitalized with influenza a and B infections. Clin Infect Dis.

[26] South East Asia Infectious Disease Clinical Research Network (2013). Effect of double dose oseltamivir on clinical and virological outcomes in children and adults admitted to hospital with severe influenza: double blind randomised controlled trial. BMJ (Clinical Research Ed).

[27] Leekha S, Zitterkopf NL, Espy MJ, Smith TF, Thompson RL, Sampathkumar P (2007). Duration of influenza A virus shedding in hospitalized patients and implications for infection control. Infect Control Hosp Epidemiol.

[28] Frank AL, Taber LH, Wells CR, Wells JM, Glezen WP, Paredes A (1981). Patterns of shedding of myxoviruses and paramyxoviruses in children. J Infect Dis.

